# Impaired glutamatergic neuron–fibroblast communication driving skin aging

**DOI:** 10.1186/s43556-026-00668-1

**Published:** 2026-11-14

**Authors:** Jun-Wei Cao

**Affiliations:** School of Life and Health Sciences, Hunan University of Science and Technology, Xiangtan, 411100 Hunan China

## Main text

A recent study published in Cell by Wang et al. identified a functional pathway through which skin-innervating glutamatergic neurons sustain dermal fibroblast function and collagen homeostasis, and showed that its age-related decline drives fibroblast senescence and skin aging in mice [1]. These findings reveal a bridge between peripheral neuronal aging and fibroblast senescence and suggest that targeting neuronal glutamate release or restoring neuron–fibroblast crosstalk could delay skin aging.

As a primary barrier organ, the skin undergoes marked structural and functional deterioration with age, manifested as collagen loss, impaired wound repair, and increased susceptibility to infection and malignancy. Accumulating evidence suggests that fibroblast senescence is a key driver of skin aging [2]. In aged skin, dermal fibroblasts accumulate in a senescent state characterized by p16 expression, a senescence‑associated secretory phenotype, and a progressive loss of collagen‑producing capacity; these senescent fibroblasts also remodel the extracellular matrix and acquire an inflammatory phenotype that further exacerbates skin aging and aging‑related dermatoses [3]. Nevertheless, the upstream regulatory mechanisms governing fibroblast senescence in the skin have remained largely unknown.

The skin is densely innervated by sensory nerve fibers that not only convey pain, temperature, itch, and chemical stimuli but also release neurotransmitters acting on other cells in the skin microenvironment; aberrant neuromodulation has been implicated in several skin conditions, including vitiligo [4]. Whether skin-innervating nerves directly orchestrate fibroblast senescence and collagen homeostasis, and whether age-associated neural changes are a cause or a consequence of skin aging, have remained unresolved.

**Fig. 1:**
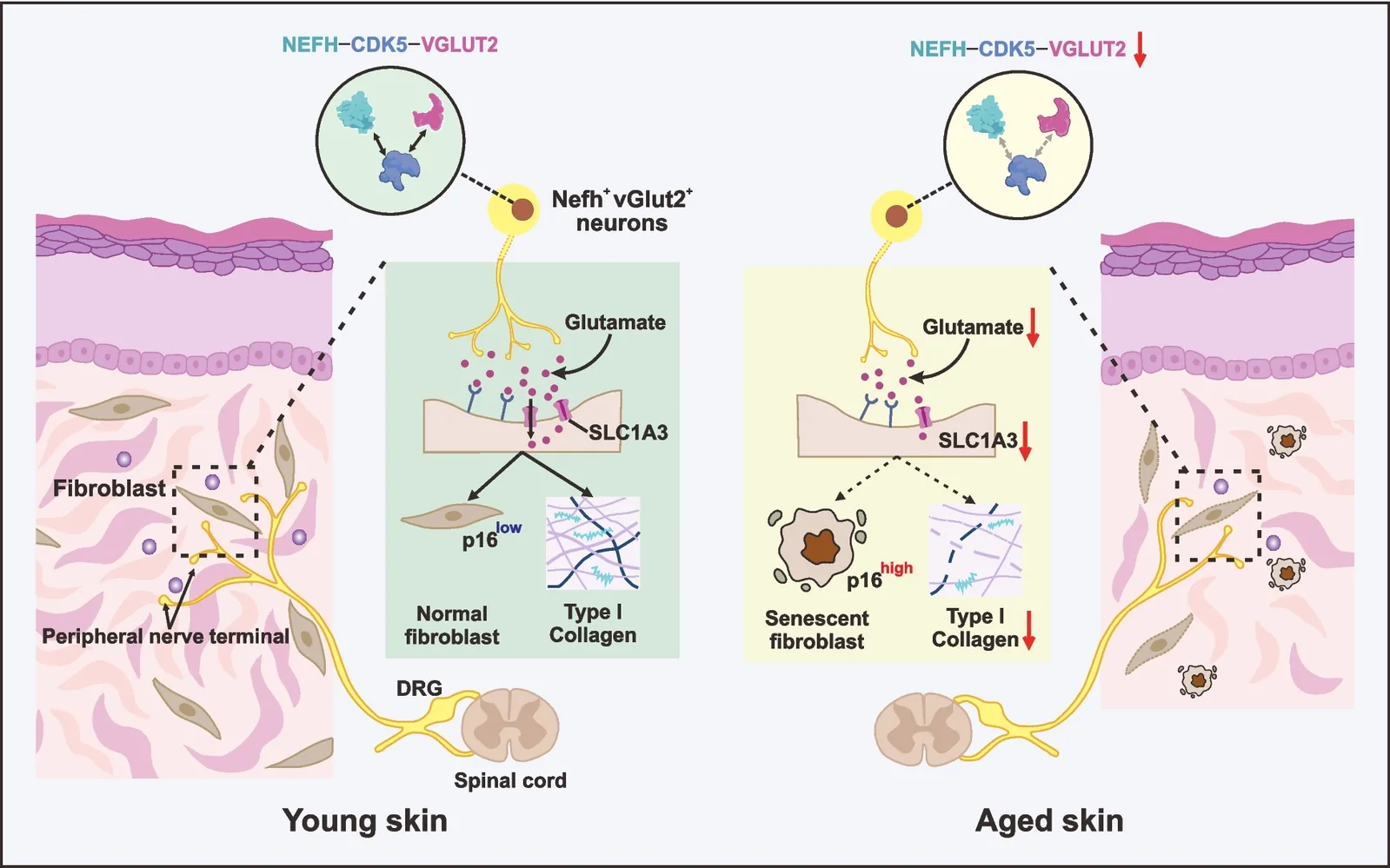
Cellular substrates and signaling pathways underlying neuromodulation of skin aging. The diagram illustrates how sensory neurons regulate dermal fibroblast function and skin aging through the neuronal NEFH–CDK5–VGLUT2 pathway, glutamate release, and fibroblast SLC1A3-dependent signaling. Left panel (young skin): NEFH, CDK5, and VGLUT2 are expressed in Nefh⁺ vGlut2⁺ neurons of the dorsal root ganglion (DRG), whose peripheral terminals innervate the skin; their interaction and functional interdependence support vesicular glutamate release. Protein associations are supported by co-immunoprecipitation and functional rescue and do not establish a simultaneous ternary NEFH–CDK5–VGLUT2 complex or direct binding among all three components. Glutamate acts on the SLC1A3 transporter on dermal fibroblasts, and SLC1A3-dependent signaling suppresses the senescence marker p16 (“p16^low^”) and promotes type I collagen production, maintaining a healthy extracellular matrix and normal fibroblast morphology. Right panel (aged skin): Reduced NEFH–CDK5–VGLUT2 pathway activity leads to decreased glutamate release and reduced SLC1A3-dependent signaling in fibroblasts (red downward arrow). Weakening of the SLC1A3-dependent signaling (thinner dashed arrows) results in decreased type I collagen production (red downward arrow) and increased p16 expression (“p16^high^”), promoting fibroblast senescence and skin aging

Wang et al. addressed this question by investigating the cellular substrates and molecular mechanisms underlying peripheral neuromodulation in skin aging [1]. The authors found that skin-innervating neurons play a key role in skin aging, since cutaneous denervation accelerated aging. Further investigations demonstrate that neurofilament heavy chain (NEFH), which is predominantly expressed in vesicular glutamate transporter 2-positive (VGLUT2^+^) glutamatergic neurons and subject to age‑related decline, serves as a molecular regulator of fibroblast senescence. Mechanistically, cyclin-dependent kinase 5 (CDK5) interacts with both NEFH and VGLUT2 to sustain glutamate release. Therefore, aging-related NEFH deficiency impairs glutamate release via the NEFH–CDK5–VGLUT2 axis, thereby driving fibroblast senescence and collagen loss. Conversely, glutamate supplementation ameliorates fibroblast senescence and improves skin aging phenotypes in a manner that depends on solute carrier family 1 member 3 (SLC1A3). Collectively, this study establishes functional neuron–fibroblast crosstalk and identifies a neuronal NEFH–CDK5–VGLUT2 → glutamate → fibroblast SLC1A3 pathway that underlies the neuromodulation of skin aging (Fig. 1).

To establish the role of skin-innervating neurons in skin aging, the authors adopted multiple cutaneous denervation models and found that denervation increased p16 expression and accelerated collagen loss—phenotypes that serve as a proxy for, but are not equivalent to, the full process of physiological skin aging. The authors then sought to identify the changes in these cutaneous neurons that drive skin aging. Using public transcriptomic datasets, the authors found that *Nefh* was among the most dramatically downregulated genes in aged human skin, and NEFH protein levels were significantly reduced in both aged human and mouse skin; NEFH expression correlated negatively with age and positively with collagen abundance. Although these human data are associative, they point to NEFH as a candidate regulator of skin aging. Single-cell transcriptomics combined with multiple neuronal labeling techniques localized NEFH predominantly to VGLUT2⁺ skin-innervating glutamatergic neurons, whose fibers contact fibroblasts spatially, consistent with direct neuron–fibroblast communication.

To confirm the role of NEFH in skin aging, the authors deleted *Nefh* in skin-innervating neurons (gene deletion, not cell ablation) and observed typical skin-aging phenotypes, including collagen depletion, p16 upregulation, and fibroblast senescence (SA-β gal staining). Further investigation revealed that *Nefh* deletion markedly reduced VGLUT2 expression and thereby likely impaired glutamate release, given that VGLUT2—a transporter encoded by *Slc17a6*—loads glutamate into synaptic vesicles. Consistent with this, tissue glutamate is reduced in aged human and mouse skin and after denervation or *Nefh* downregulation; however, tissue glutamate is not a direct measure of neuronal release, so these data support, but do not prove, impaired glutamate release. Furthermore, intracutaneous glutamate injection into *Nefh*^fl/fl^; *Vglut2*^Cre^ mice—which carry a global *Nefh* deletion rather than one restricted to skin-innervating neurons—significantly rescued NEFH deficiency-induced collagen loss and fibroblast senescence. Collectively, these results suggest that NEFH is not simply a skin aging-associated marker, but actively regulates skin aging via neuronal glutamate release.

Mechanistically, proteomic and co-immunoprecipitation analyses identified CDK5 as a NEFH-interacting protein in mouse skin that also interacts with VGLUT2. NEFH deficiency reduced neuronal CDK5 and VGLUT2 expression and impaired glutamate release, and *Cdk5* overexpression restored VGLUT2 levels and glutamate production. These interaction and rescue data support a functional NEFH–CDK5–VGLUT2 pathway governing glutamate release. Whether NEFH directly controls VGLUT2 expression or glutamate release, or acts through CDK5-dependent intermediate mechanisms, remains unresolved. Notably, in a bleomycin‑induced skin fibrosis model, CDK5 inhibition or neuronal *Nefh* deletion reduced excessive collagen deposition. This suggests that the pathway promotes collagen accumulation in a context-dependent manner—potentially beneficial in aging skin but unfavorable in fibrosis—rather than establishing an intrinsic homeostatic set point. Accordingly, although these findings support a preclinical rationale for targeting neuron–fibroblast communication in skin aging, any collagen-restoring intervention must be balanced against the risk of excessive deposition or fibrosis in susceptible contexts.

Finally, integrating a screen of glutamate receptors and transporters with fibroblast-specific labeling, the authors identified SLC1A3, a glutamate transporter rather than a canonical glutamate receptor, as highly expressed in skin fibroblasts and reduced in aged human and mouse skin. Fibroblast-specific *Slc1a3* knockout increased p16 and decreased collagen in young mice, and the anti-senescence and collagen-promoting effects of glutamate on mouse and human skin fibroblasts were largely abrogated by *Slc1a3* knockout or knockdown. Together, these results suggest that SLC1A3 is essential for fibroblasts to sense glutamate and sustain skin collagen homeostasis, although whether glutamate uptake or a downstream metabolic or signal transduction mechanism mediates these effects remains unresolved.

These findings broaden our understanding of skin aging by implicating peripheral neurons in the regulation of fibroblast function and collagen homeostasis. The strength of this study lies in shifting the focus of skin anti-aging from fibroblasts themselves to neuron–fibroblast communication. The relationship between NEFH decline and skin aging may be bidirectional. The findings of Wang et al. support a causal contribution of neuronal NEFH loss to skin aging. Conversely, aged skin typically displays reduced nerve fiber endings, raising the possibility that skin aging secondarily impairs sensory neuron function and NEFH expression. Future work will be needed to determine whether NEFH decline is primarily a driver of skin aging, a consequence of it, or both.

This study identifies NEFH⁺VGLUT2⁺ glutamatergic neurons as important regulators of fibroblast function and skin aging in mice. However, because the skin contains multiple neuronal populations and neurotransmitters [5], it remains to be determined how broadly this mechanism applies to cutaneous aging and what the relative contributions of NEFH⁺VGLUT2⁺ neurons and other neuronal populations are. Oral glutamate administration attenuated skin aging in mice, but this finding does not establish clinical efficacy or distinguish the contributions of local neuronal glutamate from those of systemic glutamate; indirect systemic mechanisms therefore remain possible, and delivery of dietary glutamate to dermal fibroblasts has not been demonstrated. Several unresolved questions merit particular attention. First, what causes the age-associated decline in NEFH levels and SLC1A3-mediated signaling? Second, how does SLC1A3 couple glutamate availability to fibroblast function? Third, can a local intervention that restores neuron–fibroblast signaling preserve skin function during aging without promoting fibrosis? Addressing these questions will be important for translating peripheral neuromodulation into safe anti-aging strategies.

Overall, this study identifies a functional neuronal pathway that links skin innervation to fibroblast function and collagen homeostasis, and indicates that skin-innervating nerve fibers—including NEFH⁺VGLUT2⁺ glutamatergic neurons—are important regulators of skin aging and skin health.

## Data Availability

Not applicable.
